# Phenotypic and Genotypic Antibiotic Resistant diarrheagenic *Escherichia coli* pathotypes isolated from Children with Diarrhea in Nairobi City, Kenya

**DOI:** 10.4314/ejhs.v30i6.5

**Published:** 2020-11

**Authors:** Mark Kilongosi Webale, Bernard Guyah, Christine Wanjala, Peter Lokamar Nyanga, Sella K Webale, Collins Abonyo, Nicholas Kitungulu, Nathan Kiboi, Nancy Bowen

**Affiliations:** 1 School of Health Sciences, Kirinyaga University, Kutus, Kenya; 2 School of Public Health, Maseno University, Kenya; 3 School of Public Health, Masinde Muliro University of Science and Technology, Kakamega, Kenya Unit, Ministry of Health, Nairobi city, Kenya; 4 Disease Surveillance and Response; 5 School of Natural Sciences, Masinde Muliro University of Science and Technology, Kenya; 6 School of Pure and Applied Sciences, Kenyatta University, Kenya; 7 National Public health Laboratories, Ministry of Health, Nairobi city, Kenya

**Keywords:** Phenotypic, Genotypic, Antibiotic Resistant, Escherichia coli pathotypes, Diarrhea

## Abstract

**Background:**

The marked genome plasticity of diarrheagenic Escherichia coli promotes emergence of pathotypes displaying unique phenotypic and genotypic resistance. This study examined phenotypic and genotypic antibiotic resistant diarrheagenic Escherichia coli pathotypes among children in Nairobi City, Kenya.

**Methods:**

In a cross-sectional study, diarrheagenic Escherichia coli pathotypes were isolated from stool samples and their phenotypic and genotypic resistance against eight antimicrobial agents assayed.

**Results:**

Diarrheagenic Escherichia coli was detected in 136(36.4%) children. Most of diarrheagenic Escherichia coli that were resistant to ampicillin, ceftriaxone, streptomycin, gentamycin, ciprofloxacin, chloramphenicol, erythromycin and tetracycline, harbored citm, bla CMY, aadA1, aac(3)-IV, qnr, catA, ere(A) and tet(A) corresponding resistant genes.

**Conclusion:**

Antimicrobial-resistant genes are highly prevalent among phenotypic resistant ETEC pathotypes indicating a possibility of horizontal gene transfer in spreading antibiotic resistant genes among E. coli pathotypes.

## Introduction

There is a worldwide concern about the rise and spread of bacterial resistance to commonly prescribed antimicrobial agents. A United Nation's report revealed that antibiotic resistance cause at least 700,000 deaths globally a year currently, and the figure could increase up to 10 million deaths globally by 2050, without a sustained effort to contain antimicrobial resistance (1). Meanwhile, the World Health Organization forewarns the severity of antibiotic resistance, stating that “it threatens the achievements of modern medicine, a post-antibiotic era, in which common infections and minor injuries can kill, is a very real possibility for the 21st century” (2). In this regard, programs for monitoring antimicrobial resistance have been established in many countries worldwide, including the antimicrobial resistance surveillance program of the National Public Health Laboratories (NPHLs) and Kenyan Medical Research Institute (KEMRI) in Kenya (2).

Diarrheal illnesses are a severe public health problem and a major cause of morbidity and mortality in infants and young children globally (3). Diarrhegenic *E. coli* involved in diarrheal diseases is one of the most important of the various etiological agents of diarrhea (3). In Kenya, over 15% infectious diarrhea cases present in health facilities (4,5) but only half of health facilities are able to detect and diagnose a pathogen, which may be due to lack of or inadequate diagnostic capacity (6). Thus, indiscriminate antibiotic treatment is crucial for weak individuals with severe dysentery and nondysentery infection without secondary criteria for bacterial infection (4,7) increasing selection pressure of antibiotic resistant strains and decreasing the effectiveness of antibiotics (8). With an exception of few studies (9–12) previous studies reported phenotypic resistance without assessing genetic changes associated with resistance in diarrhegenic and uropathogenic *Escherichia coli* to commonly prescribed antibiotics in Kenya (13–20). Genetic changes associated with phenotypic resistance to quinolones, gentamycin, tetracycline, Sulfonamide, Trimethoprim and beta-lactams has been investigated in diarrhegenic *Escherichia coli* (10–12) while phenotypic expression of quinolones and beta-lactams resistant genes have been investigated in uropathogenic *Escherichia coli* (9) in kenya. To our knowledge, no study investigated erythromycin, chloramphenicol and streptomycin resistant genes in diarrhegenic *Escherichia coli* isolated from humans in Kenya.

According to the group of virulence determinants acquired, specific combinations were formed determining the currently known diarrheagenic *Escherichia coli* pathotypes and comprises of six groups: enteropathogenic *E. coli* (EPEC), enterohemorrhagic (Shiga toxin-producing) *E. coli* (EHEC/STEC), enteroaggregative *E. coli* (EAEC), enterotoxigenic *E. coli* (ETEC), enteroinvasive *E. coli* (EIEC) and diffuse adhering/diffusely adherent E. coli (DAEC). Until now, there is limited information about the distribution of phenotypic and genotypic antibiotic resistance across these *Escherichia coli* pathotypes in humans (21–23). A previous study in Northern Iran (Tehran City), indicated high phenotypic resistance frequency of STEC in a population of EPEC, STEC, EAEC and ETEC infected diarrheic children (22) while a study in Western Iran reported high phenotypic resistant rates of EHEC in a population of EPEC, STEC and EHEC infected children (23) suggesting that *Escherichia coli* phenotypic resistance is highly polymorphic attributed to genome plasticity of *E. coli* accelerating emergence of pathotypes displaying unique antibiotic phenotypic resistance (24–26). In India, EPEC were found to harbor higher number of antibiotic resistant genes in a population of EAEC, ETEC, EIEC and EHEC isolates from diarrheic children (21). Since there are significant differences in antibiotic use between countries indicating that some countries are probably overusing antibiotics (27,28) which may drive development of antimicrobial resistance in genetically and geographically diverse diarrheagenic *Escherichia coli* pathotypes (8,24–26), to our knowledge, no study has determined rates of antimicrobial resistant genes concurrently in EAEC, ETEC, EIEC and EHEC clinical isolates from humans in Kenya. This study, therefore, determined phenotypic and genotypic antibiotic resistance of diarrheagenic *Escherichia coli* pathotypes isolated from children with diarrhea in Nairobi City, Kenya

## Methods

**Study site, design and population**: Detailed description of the study site, design and population is presented here (13). Briefly, this was a cross-sectional study targeting diarrheic children <5 years, seeking treatment for diarrhea at Mbagathi Hospital, Nairobi City, Kenya. A total of 374 children with diarrhea were enrolled into the study. Diarrhea was defined according to World Health Organization (WHO) guidelines (29). Demographic and clinical information of these study participants were collected using a questionnaire. Stool microbiology tests were performed within two hours of sample collection. Stool samples of children who had received antibiotics were excluded from the study.

**Bacteriological procedures**: Identification of *E.coli* species was performed by following the WHO recommendations (30). Briefly, stool samples were plated on MacConckey Agar (MCA), Xylose lysine Deoxycholate (XLD), and sorbitol MacConkey agar (SMAC) and incubated at 37°C overnight. Complete biochemical identification was used to confirm the identity of the cultured organism. DNA from cultured *E. coli* isolates was extracted using QIAamp® DNA Mini Kit (QIAGEN GmbH, Hilden, Germany) according to the manufacturer's recommendations. Conventional polymerase chain reaction (PCR) assay was used to detect diarrhegenic *E. coli* pathotypes based on specific virulence genes as previously described (13).

**Phenotypic Antimicrobial Resistance**: Antimicrobial susceptibility testing was carried out on Muller-Hinton agar with antibiotic discs by the disc diffusion method based on guidelines adopted from Clinical and Laboratory Standards Institute (CLSI) (31). Antibiotics discs of ampicillin, ceftriaxone, streptomycin, gentamycin, ciprofloxacin, chloramphenicol, erythromycin and tetracycline were tested.

**Detection of antibiotic resistant genes**: DNA from cultured isolates was extracted using QIAamp® DNA Mini Kit (QIAGEN GmbH, Hilden, Germany) according to the manufacturer's recommendations. The isolates were grouped on the basis of resistance phenotype and determined for the presence of corresponding antibiotic resistance genes. The presence of resistance genes to ampicillin: *citm*, ceftriaxone: *bla CMY*, streptomycin: aadA1, gentamycin: *aac(3)-IV*, ciprofloxacin: *qnr*, chloramphenicol: *catA1*, erythromycin: *ere(A)* and tetracycline: *tet(A)* were detected by PCR. The primers sequences and the amplicon sizes are listed in Table 1. Amplified samples were analyzed by electrophoresis in 1.5% agarose gel and stained by ethidium bromide.

**Table 1:** Primers used for detection of antimicrobial resistant genes

Antibiotic type	Antibiotic resistant gene	Primer sequence	Amplicon size
Ampicillin	*citm*	F: TGG CCA GAA CTG ACA GGC AAA R: TTT CTC CTG AAC GTG GCT GGC	462
Ceftriaxone	*β-lactamase* encoding cephalosporin resistance (*bla* *CMY*)	F: TGGCCAGAACTGACAGGCAAA R: TTTCTCCTGAACGTGGCTGGC	462
Streptomycin	*Adenylyl transferases* *(aadA1)*	F: TATCCAGCTAAGCGCGAACT R: ATTTGCCGACTACCTTGGTC	447
Gentamycin	*Aminoglycoside* *acetyltransferases (aac(3)-IV)*	F: CTTCAGGATGGCAAGTTGGT R: TCATCTCGTTCTCCGCTCAT	286
Ciprofloxacin	*quinolone resistance protein* *(qnr)*	R: GGGTATGGATATTATTGATAAAG R: CTAATCCGGCAGCACTATTTA	670
Chloramphenicol	*Acetyltransferases (catA1)*	F: AGTTGCTCAATGTACCTATAACC R: TTGTAATTCATTAAGCATTCTGCC	547
Erythromycin	*Erythromycin esterase* *(ere(A))*	F: GCCGGTGCTCATGAACTTGAG R: CGACTCTATTCGATCAGAGGC	419
Tetracycline	*Efflux pump resistance* *(tet(A))*	F: GGTTCACTCGAACGACGTCA R: CTGTCCGACAAGTTGCATGA	577

Primers used in the current study were adopted from previous studies (40, 41).

**Data analysis**: Statistical analyses were performed using SPSS version 19.0 for Windows (IBM SPSS Statistics for Windows, Version 19.0. Armonk, NY: IBM Corp.). Descriptive statistics; namely, frequencies and percentages were used to present demographic and clinical data, and phenotypic and genotypic frequencies.

**Ethical considerations**: Ethical approval for this study was granted by Kenyatta National Hospital/University of Nairobi (KNH-UoN) Ethics and Research Committee and was conducted according to Helsinki declarations (32). Written informed consent was sought from either parent or guardian of each child. Diarrheic children were treated by clinicians according to the World Health Organization (WHO) guidelines for treatment of diarrhea in children (29). All study participants' information and test results were confidentially kept. The results of bacterial cultures were used in clinical management of study participants.

## Results

In this study, a total of 374 children were recruited; diarrheagenic *Escherichia coli* was successfully isolated in 136(36.4%) children. The demographic and clinical information of the 136 children infected with diarrheagenic *Escherichia coli* is presented in Table 2. Age distribution showed that, 63(46.3%) were within the age group between 1 and 36 months, and 73 (53.7%) children were between 37 and 60 months. The overall gender distribution was 55(40.4%) females and 81(59.6%) males. Guardians of 126(92.6%) and 10(7.4%) children reported using piped and borehole water, respectively. In addition, 69(50.7%) reported treating drinking water.

**Table 2:** Demographic and clinical information of study participants

Characteristics	Number (%)
**Age in months**	
1–36	63(46.3)
37–60	73(53.7)
**Gender**	
Female	55(40.4)
Male	81(59.6)
**Source of water**	
Piped water	126(92.6)
Borehole	10(7.4)
Water treatment	69(50.7)
**Body temperature**	
<38.0	21(15.4)
≥ 38.0	115(84.6)
**Duration of diarrhea**	
1–3	87(64.0)
4–6	24(17.6)
≥7	25(18.4)
**Symptoms**	
Vomiting	127(93.4)
Fever	118(86.8)
Abdominal cramp	129(94.9)
Headache	33(24.3)
Nausea	44(32.4)
Appetite loss	131(96.3)
Sunken eyeball	116(85.3)
Dry tongue	47(34.6)
Reduced skin elasticity	69(50.7)
***E. coli* pathotype**	
EAEC	78(57.4)
EPEC	2(1.5)
ETEC	15(11.0)
EIEC	38(27.9)
EAEC/ETEC	2(1.5)
EAEC/EPEC/ETEC	1(0.7)

Temperature of <38.0°C and ≥ 38.0°C was recorded in 21(15.4%) and 115(84.6%) children, respectively. In this study, 87(64.0%), 24(17.6%) and 25(18.4%), respectively, reported having diarrhea for 1–3, 4–6 and ≥7 days. Vomiting was reported in 127(93.4%), fever in 118(86.8%), abdominal cramp in 129(94.9%), headache in 33 (24.3%), nausea in 44 (32.4%), and appetite loss in 131(96.3%) children. Clinical diagnosis of dehydration revealed that 116(85.3%) had sunken eyeballs, 47(34.6%) children had dry tongue and 69(50.7%) had reduced skin elasticity. Diarrheagenic *Escherichia coli* pathotyping showed that 78(57.4%), 2(1.5%), 15(11.0%) and 38(27.9%) were infected with EAEC, EPEC, ETEC and EIEC pure strains, while mixed pathotype infection was detected in 2(1.5%) children for EAEC/ETEC and 1(0.7%) child for EAEC/EPEC/ETEC.

**Antimicrobial resistant phenotypes and genes of diarrheagenic *Escherichia coli***: Antimicrobial resistant phenotypes and genes of diarrheagenic E. coli are presented in Table 3. A total of 71(53.4%), 17(12.8), 89(66.9), 91(68.4), 40(30.1), 87(65.4), 12(9.0) and 111(83.5) diarrheagenic *Escherichia coli* isolates identified as phenotypic resistant to ampicillin, ceftriaxone, streptomycin, gentamycin, ciprofloxacin, chloramphenicol, erythromycin and tetracycline, respectively, 70(98.6%), 15(88.2%), 83(93.3), 60(65.9%), 38(95.0%), 85(87.6%), 11(91.7%) and 102(91.9%) contained *citm, bla CMY, aadA1, aac(3)-IV, qnrA1, catA, ere(A)* and *tet(A)* corresponding antibiotic resistant genes.

**Table 3:** Antimicrobial resistant phenotypes and genes of diarrheagenic *Escherichia coli*

Antibiotic	Resistant phenotype (n)	Resistant Gene	Number (%)
Ampicillin	71(53.4)	*citm*	70 (98.6)
Ceftriaxone	17(12.8)	*bla CMY*	15 (88.2)
Streptomycin	89(66.9)	*aadA1*	83 (93.3)
Gentamycin	91(68.4)	*aac(3)-IV*	60 (65.9)
Ciprofloxacin	40(30.1)	*qnr*	38 (95)
Chloramphenicol	87(65.4)	*catA1*	85 (87.6)
Erythromycin	12(9.0)	*ere(A)*	11 (91.7)
Tetracycline	111(83.5)	*tet(A)*	102 (91.9)

Data are presented as number and proportions (%) of study participants. *bla SHV*, β-lactamase encoding penicillin resistance. *bla CMY*, β-lactamase encoding cephalosporin resistance. *aadA1*, adenylyl transferases. *aac(3)-IV*, aminoglycoside acetyltransferases. *qnr*, quinolone resistance protein. *catA1*, acetyltransferases. *ere(A)*, erythromycin esterase. *tet(A)*, efflux pump resistance.

**Phenotypic and genotypic antibiotic resistance of *E. coli* pathotypes**: Phenotypic resistant rate of ampicillin was 53.8%, 100.0%, 40.0% and 55.3% while ceftriaxone resistance rate was reported 10.3%, 0.0%, 13.3% and 18.4% in EAEC, EIEC, EPEC and ETEC pathotypes, respectively. Streptomycin resistance rate was 67.9%, 100.0%, 66.7%, and 63.2% while that of gentamycin was 65.4%, 100.0%, 60.0%, and 76.3% in EAEC, EIEC, EPEC and ETEC pathotypes, respectively. Ciprofloxacin resistant rates were 25.6%, 50.0%, 20.0%, and 42.1% while chloramphenicol resistant rates were 59.0%; 50.0%, 66.7% and 78.9%, 25.6%, 50.0%, 20.0%, and 42.1%, in EAEC, EIEC, EPEC and ETEC pathotypes, respectively. Erythromycin resistance rate was 9.0%, 50.0%, 0.0% and 10.5% of while tetracycline resistance rate was 8.8%, 100.0%, 93.3% and 84.2% EAEC, EIEC, EPEC and ETEC pathotypes, respectively. The phenotypic resistant isolates were assayed for the presence of resistant genes. *Citm* gene was detected in 52.6%%, 100.0%%, 40.0%, and 55.3% while *bla CMY* was detected in 10.3%, 0.0%, 13.3%, and 13.2% in EAEC, EIEC, EPEC and ETEC pathotypes, respectively. The rate of *aadA1* was 61.5%, 100.0%, 66.7% and 60.5% while that of *aac(3)-IV* was 39.7%, 0.0%, 60.0% and 52.6% in EAEC, EIEC, EPEC and ETEC pathotypes, respectively. Prevalence of *qnrA1* was 24.4%, 50.0%, 20.0% and 39.5% while that of *catA* was 60.3%, 50.0%, 66.7% and 71.1% in EAEC, EIEC, EPEC and ETEC pathotypes, respectively. Prevalence of *ere(A)* was 9.0%, 0.0%, 0.0% and 10.5% while that of *tet(A)* was 71.8%, 50.0%, 93.3% and 81.6% in EAEC, EIEC, EPEC and ETEC pathotypes, respectively.

## Discussion

Diarrheagenic *Escherichia coli* is a major etiology of bacterial diarrhea globally (3). Even though antibiotics have been used to control *E. coli* infections, the marked genome plasticity of *E. coli* has allowed the emergence of pathotypes displaying unique virulence and antimicrobial resistance genes (33). Furthermore, the prevalence of *E. coli* pathotypes and their antimicrobial resistance differ geographically (25). Thus, assessing the diversity and distribution of antibiotic resistant genes in a population of *E. coli* pathotypes represents a more detailed and potentially useful additional tool for improving our understanding of antimicrobial resistance epidemiology.

Dysentery, non-dysentery infections and other clinical complications of infections are serious among Kenyans presenting in health facilities without capacity to diagnose and detect bacterial pathogens, compelling clinicians to consider the provision of empirical antibiotic therapy (4,7). In addition, Kenya has no legislation for controlling antibiotic use in animals; further pressure is applied on antibiotic use as growth promoters and not for the treatment of infections of farm animals (34). At the same time, although the purchase of antibiotics from retail pharmacies without a prescription is forbidden by Kenya's Pharmacy and Poisons Board (35), over-the-counter sale of antimicrobials without a prescription is possible and may aggravate antibiotic resistance and spread resistant strains (36,37). Because inappropriate antibiotic use selects antibiotic resistance, it was not surprising that our study found high phenotypic antibiotic resistance rates to ampicillin, streptomycin, gentamycin, ciprofloxacin, chloramphenicol and tetracycline suggesting that these six drugs should not be used as a first-line therapeutic drug for diarrheagenic *Escherichia coli*. After phenotypic screening, genes associated with antimicrobial resistance were determined by polymerase chain reaction (PCR). In this study, resistance genes detected were *citm, bla CMY, aadA1, aac(3)-IV, qnrA1, catA, ere(A)* and *tet(A)* for ampicillin, ceftriaxone, streptomycin, gentamycin, ciprofloxacin, chloramphenicol, erythromycin and tetracycline, respectively. Interestingly, a proportion of phenotypic resistant diarrheagenic *Escherichia coli* did not harbor these antimicrobial resistant genes while the isolates were fully resistant, suggesting a possibility of existence of other antimicrobial resistant genes and mechanisms of causing antimicrobial resistance such as efflux pumps. This is the first study to investigate *citm, aadA1, aac(3)-IV, catA,* and *ere(A)* in diarrheagenic *Escherichia coli* samples isolated from human beings in Kenya. The observations of the present study are similar to previous studies in Kenya that detected *blaCMY, qnrA and qnrB* (10,11), and *tetA* in *E. coli* isolated from stool samples (12) and *bla CMY, qnrA* and *qnrB* in *E. coli* isolated from urine samples (9). In addition, these observations are consistent with previous studies that detected *aadA1, tetA, qnr, aac(3)-IV, citm* and *cat1* for streptomycin, tetracycline, ciprofloxacin, gentamycin, ampicillin and chloramphenicol resistance genes, respectively, in *E. coli* isolated from stool samples among pediatric patients younger than five years in Iran (38). Possible explanation for the persistence of resistance to these antibiotics includes the frequent co-existence of resistant genes on large transferable plasmids (39) after a possible antibiotic selection pressure (8). Hence, widespread public health education and supervision of the sales and prescription of antibiotics in retail pharmacies and hospitals are needed, to preserve the effectiveness of remaining antibiotics.

To our knowledge, the present study is the first in Kenya that investigated simultaneously the presence of phenotypic and genotypic resistance in four diarrheagenic *Escherichia coli* (EAEC, EIEC, EPEC and ETEC) pathotypes. In our study, there was a high incidence of phenotypic resistant isolates of ETEC to ampicillin, gentamycin, ciprofloxacin, chloramphenicol and tetracycline. A study in Northern Iran (Tehran City) that concurrently isolated EPEC, STEC, EAEC and ETEC from patients with diarrhea indicated high phenotypic resistance frequencies of STEC to ampicillin, tetracycline, streptomycin and chloramphenicol (22), while another study in Western Iran that concurrently isolated EPEC, STEC and EHEC from diarrheic patients revealed high phenotypic resistant rates in EHEC to ampicillin, tetracycline and ciprofloxacin (23). Consistent with phenotypic resistance, the frequency of Ampicillin (*citm)*, gentamycin (*aac(3)-IV),* ciprofloxacin (*qnr)*, chloramphenicol (*catA),* tetracycline *(tetA)* resistant genes was high in ETEC. This suggest that ETEC is a reservoir of antimicrobial resistant genes which can easily get transferred among the diarrehgenic *E. coli* community via horizontal gene transfer. This hypothesis is rainforced by the presence of EAEC/ETEC and EAEC/EPEC/ETEC hybrids in this study (24) thus exhibiting the phenomenon of antibiotic resistance genes (*citm, blaCMY, aadA1, aac3, qnr, catA, ere(A), tetA*) detected in EAEC, EIEC and EPEC isolates in this study. A study in India that concurrently isolated ETEC, EIEC, EAEC and EHEC from adults and children patients detected *tetA* in EIEC, *aac3* in ETEC, EIEC and EAEC, *catA* in ETEC, EIEC and EAEC, *aad* in ETEC, EAEC and EHEC and *qnrS* but not *qnrA* and *qnrC* in ETEC, EIEC, EAEC and EHEC pathotypes (21). The marked genome plasticity of diarrheagenic *E. coli* accelerates the adaptation of these pathotypes to antibiotic environment (24, 26). This allow emergence of strains displaying unique phenotypic and genotypic antimicrobial resistance patterns under selection pressure of antibiotics (24, 26). Taken together, ETEC is a reservoir of antibiotic resistant gene which can be horizontally transferred to other diarrheagenic *E. coli* pathotypes among diarrheic children in Kenya. Therefore, it is imperative to develop strategies to control the spread of resistant strains.

It is important to note that the present study had limitations. Sources of these antimicrobial resistant genes were not investigated. Study participants were recruited within the hospital; hence, the prevalence of phenotypic and genotypic antimicrobial resistance rates does not represent community prevalence. Due to financial constraints, we were not able to study more antibiotics and antimicrobial resistant genes than what we have done, even though multiple genes can confer resistance to antibiotics. Also, we did not find the association between phenotypic and genotypic resistance.

We observed that DEC is highly resistant to ampicillin, streptomycin, gentamycin, ciprofloxacin, chloramphenicol and tetracycline and the resistance is driven by antimicrobial resistant genes. ETEC and EAEC play an important role as a potential reservoir of these antibiotic resistant genes, thus illustrating the importance of horizontal gene transfer.

## Figures and Tables

**Table 4:** Phenotypic and genotypic antibiotic resistance of *E. coli* pathotypes

Antibiotic Resistance	EAEC (n=78)	EIEC (n=2)	EPEC (n=15)	ETEC (n=38)
**Phenotypic resistance**				
Ampicillin	42(53.8)	2(100.0)	6(40.0)	21(55.3)
Ceftriaxone	8(10.3)	0(0.0)	2(13.3)	7(18.4)
Streptomycin	53(67.9)	2(100.0)	10(66.7)	24(63.2)
Gentamycin	51(65.4)	2(100.0)	9(60.0)	29(76.3)
Ciprofloxacin	20(25.6)	1(50.0)	3(20.0)	16(42.1)
Chloramphenicol	46(59.0)	1(50.0)	10(66.7)	30(78.9)
Erythromycin	7(9.0)	1(50.0)	0(0.0)	4(10.5)
Tetracycline	63(8.8)	2(100.0)	14(93.3)	32(84.2)
**Genotypic resistance**				
*citm*	41(52.6)	2(100.0)	6(40.0)	21(55.3)
*bla CMY*	8(10.3)	0(0.0)	2(13.3)	5(13.2)
*aadA1*	48(61.5)	2(100.0)	10(66.7)	23(60.5)
*aac(3)-IV*	31(39.7)	0(0.0)	9(60.0)	20(52.6)
*qnr*	19(24.4)	1(50.0)	3(20.0)	15(39.5)
*catA1*	47(60.3)	1(50.0)	10(66.7)	27(71.1)
*ere(A)*	7(9.0)	0(0.0)	0(0.0)	4(10.5)
*tet(A)*	56(71.8)	1(50.0)	14(93.3)	31(81.6)

Data are presented as number and proportions (%) of study participants. *aadA1*, adenylyl transferases. *aac(3)-IV*, aminoglycoside acetyltransferases. *qnr*, quinolone resistance protein. *catA1*, acetyltransferases. *ere(A)*, erythromycin esterase. *tet(A)*, efflux pump resistance.
